# Association between lactate-to-albumin ratio and 28-days all-cause mortality in patients with acute pancreatitis: A retrospective analysis of the MIMIC-IV database

**DOI:** 10.3389/fimmu.2022.1076121

**Published:** 2022-12-14

**Authors:** Qiang Liu, Hai-Lun Zheng, Man-Man Wu, Qi-Zhi Wang, Shan-Jun Yan, Meng Wang, Jiao-Jiao Yu, Da-Peng Li

**Affiliations:** Department of Gastroenterology, The First Affiliated Hospital of Bengbu Medical College, Bengbu, Anhui, China

**Keywords:** lactate/albumin ratio, acute pancreatitis, all-cause mortality, 28-days, prognosis, cohort

## Abstract

**Objective:**

The Lactate-to-Albumin Ratio (LAR) has been applied as a new predictor in sepsis, heart failure, and acute respiratory failure. However, the role of LAR in predicting all-cause mortality in patients with acute pancreatitis has not been evaluated. Therefore, this study aimed to elucidate the correlation between LAR and 28-d all-cause mortality in patients with Acute Pancreatitis (AP).

**Methods:**

This study is a retrospective cohort study with the data from the MIMIC-IV (v1.0) database. We included adult patients with acute pancreatitis who were admitted to the intensive care unit in the study. The primary outcome was to evaluate the ability of LAR to predict death at 28-d of hospital admission in patients with AP.

**Results:**

A total of 539 patients with acute pancreatitis were included in this study. They were divided into a survival group (486 patients) and a death group (53 patients) according to whether they survived within 28-d of admission, and the mortality rate of patients within 28-d of admission was 9.8%. LAR was shown to be an independent predictor of all-cause mortality within 28-d of admission in patients with AP by multivariate COX regression analysis (HR, 1.59; 95% CI, 1.23 - 2.05; P < 0.001). the Area Under the Curve (AUC) value for LAR was 74.26% (95% CI: 67.02% - 81.50%), which was higher than that for arterial blood lactate (AUC = 71.25%) and serum albumin (AUC = 65.92%) alone. It was not inferior even when compared to SOFA (AUC = 75.15%). The optimal cutoff value for separating the survival and death groups according to Receiver Operating Characteristic (ROC) was found to be 1.1124. plotting Kaplan-Meier analysis with this cutoff value showed that patients with LAR ≥ 1.1124 had significantly higher all-cause mortality within 28-d of admission than those with LAR < 1.1124 (P < 0.001). The final subgroup analysis showed no significant interaction of LAR with each subgroup (P for interaction: 0.06 - 0.974).

**Conclusion:**

LAR can be used as an independent predictor of all-cause mortality in AP patients within 28-d of admission, with superior prognostic performance than arterial blood lactate or serum albumin alone.

## 1 Introduction

Acute pancreatitis (AP) admissions are one of the most common causes of hospitalization for gastrointestinal disorders, with an annual incidence of 13-45 cases per 100,000 for this disease (1). AP is an flammatory state of the pancreas in which initial disintegration of the alveolar cells and activation of pancreatic proteases leads to local injury, which with progression can cause systemic inflammatory response syndrome and even organ failure. The prognosis of AP depends on the severity of the disease, with approximately 75-80% of patients progressing slowly and being cured by intravenous fluid therapy and supportive care alone (2, 3). However, about 20% of patients progress to moderate or severe acute pancreatitis with critical conditions such as pancreatic or peripancreatic tissue necrosis and organ failure, with an overall mortality rate of 20% to 40% (4, 5). Therefore, early assessment of disease severity and preparation of interventions to reduce mortality is essential. Current scores that can assess the severity of AP include Ranson criteria (6), Balthazar grade (7), Acute Physiology and Chronic Health Evaluation II (APAChE-II) (6), Bedside Index for Severity in AP (BISAP) (8), etc. These scores usually require the collection of multiple indicators to complete the assessment of the patient’s condition, which increases the risk of death in some patients by missing the optimal time for treatment. For this reason, a simple, cost-effective and high-sensitivity indicator to forecast the severity of acute pancreatitis is a matter of urgent necessity.

It has been demonstrated that lactate, as a product of anaerobic metabolism, is reflective of tissue hypoperfusion and the seriousness of cellular hypoxia. It is as well able to predict organ failure and the mortality rate in critically ill patients (9, 10). However, liver dysfunction or abnormal proteolytic metabolism and metformin intake can result in abnormal lactate levels as well (11, 12). Therefore, the single use of lactate level prediction does not ensure a credible consequence. Meanwhile, we found in previous studies that albumin acts as a negative acute phase reactant, and it has been shown to correlate with inflammation severity, disease prognosis and mortality. In physiological mechanisms, serum albumin plays various roles as extracellular antioxidant, buffer, immunomodulator, detoxifier and transporter protein in plasma (13, 14). However, the patient’s nutritional status or chronic inflammation can also affect albumin levels. Therefore, prediction based on albumin levels alone may also have limitations (15). Several studies have evaluated the validity of the ratio of lactate to albumin (LAR) as a predictor in diseases such as infectious shock or severe sepsis and cardiac arrest (16–18). However, it is unclear about the relationship between LAR and mortality in patients with AP. Therefore, we obtained information on hospitalizations of patients with AP admitted between 2008-2019 through the Medical Information Mart for Intensive Care IV version 1.0 [MIMIC-IV (v1.0)] database, which aimed to analyze the relationship between LAR and acute all-cause mortality within 28-d hospital of admissions in patients with AP. And the SOFA score was used as an indicator of the severity of disease in the included population.

## 2 Methods

### 2.1 Database introduction

The data in this study are all obtained from the MIMIC-IV (v1.0) database, which is a large, publicly accessible database that was developed and managed by the MIT Computational Physiology Laboratory (https://physionet.org/content/mimiciv/1.0/). This database covers information on all patients admitted to the Beth Israel Deaconess Medical Center (BIDMC) during the years from 2008 to 2019. Each patient’s length of stay, laboratory tests, medication treatment, vital signs and other comprehensive information is recorded. And for the protection of patient privacy, all personal information is de-identified, with random codes used to replace patient identification, therefore we do not need informed consent and ethical approval of patients. The MIMIC-IV (v1.0) database can be downloaded from the PhysioNet online forum (https://physionet.org/). To access this database, the first author of this study, Qiang Liu, completed the Collaborative Institutional Training Initiative (CITI) course and passed both the “Conflicts of Interest” and “Data or Specimens Only Research” exams (ID: 50082616). The research team was finally qualified to use the database and extract data.

### 2.2 Population selection criteria

**Figure 1:**
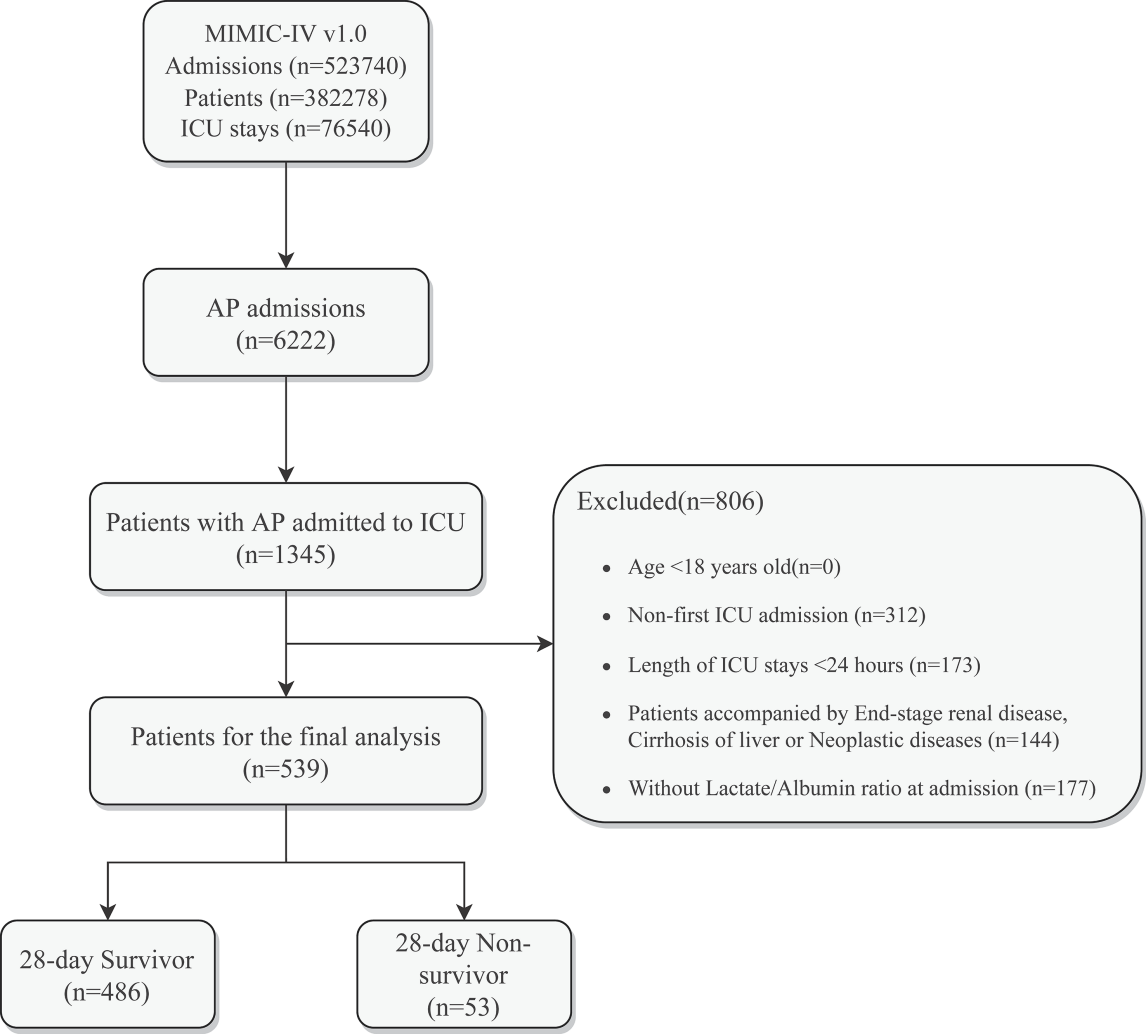
Schematic diagram of study sample selection steps. MIMIC, Medical Information Mart for Intensive Care; AP, Acute Pancreatitis; ICU, intensive care unit; LAR, Lactate/Albumin Ratio.

A total of 523,740 admissions were recorded in the MIMIC-IV database, of which 76,540 were admitted to the ICU. Hospital admission information for patients with AP was extracted according to International Classification of Diseases, 9th Revision (ICD - 9) code 577.0 and International Classification of Diseases, 10th Revision (ICD-10) codes K85-K85.92, with a total of 6222 patients included, of whom 1345 had been admitted to the ICU. After further screening, patients who meet the following criteria will be excluded (1): patients younger than 18 years of age at the time of the first admission; (2) patients admitted repeatedly for acute pancreatitis, for whom only the first admission data were retained; (3) patients who stayed in the ICU for less than 24 hours; (4) patients admitted with end-stage renal disease, cirrhosis or malignancy; (5) patients without recorded blood lactate and serum albumin data within 24 hours of admission. Ultimately, 539 patients were enrolled in this study (**Figure 1**).

### 2.3 Data extraction

The LAR was chosen as the primary study variable. Blood lactate concentration and serum albumin were measured for the first time after admission in order to minimize the interference of lactate and albumin values caused by subsequent treatment. Extraction of potential confounders included Demographics (Age, Gender, Race), Vital Signs (Heart Rate, Systolic Blood Pressure, Diastolic Blood Pressure, Mean Arterial Pressure, Respiratory Rate), Clinical Treatment (Vasopressin, Octreotide, Statins, Betablockers, and Metformin use, Mechanical Ventilation, Continuous Renal Replacement Therapy, Endoscopic Retrograde Cholangiopancreatography), Comorbidities (Acute Kidney Injury, Sepsis, Respiratory Failure, Heart Failure, Atrial Fibrillation, Hypertension, Diabetes, Obesity), Laboratory Indicators (Red Blood Cells, White Blood Cells, Erythrocyte Distribution Width, Platelets, Hemoglobin, Lymphocyte percentage, Erythrocyte Specific Volume, Total Bilirubin, Glutathione, Serum Glucose, Serum Creatinine, Blood Urea Nitrogen, Anion Gap, Prothrombin Time, International Normalized Ratio, Serum Potassium, Serum Sodium, Serum Calcium), and Sequential Organ Failure (SOFA). The data extraction tool used PostgresSQL software (v13.7.1) and Navicate Premium software (version 15) to extract data through a running Structured Query Language (SQL). All code used for the Calculation of Demographic Characteristics, Laboratory Indicators, Comorbidities and Severity Scores were obtained from the GitHub website (GitHub - MIT-LCP/mimic-iv: Deprecated. For the latest MIMIC-IV code, please refer to: https://github.com/MIT-LCP/mimic-code).

### 2.4 Grouping and endpoint events

In this study, patient subgroups were divided into a 28-d admission survival group (n=486) and a 28-d admission death group (n=53). The primary study endpoint was death from any cause within 28-d of admission. 28-Days All-Cause Mortality was defined as the ratio of the total number of deaths from all causes during a 28-d hospital stay to the average population of that population during the same period.

### 2.5 Management of missing data and outliers

To avoid bias, variables are excluded when they have more than 15% missing values, such as HbA1c, ipid profile, C-reactive protein, high sensitivity CRP, Aspartate aminotransferase, and patient height or weight. Variables with missing values greater than 5% and less than 15% (Total bilirubin, Lymphocyte percentage, PT, INR) are processed using multiple interpolations (MI) (19) to select the relative optimal set of data to fill in the missing values. On the other hand, variables with missing values less than 5% (Heart rate, Systolic Blood Pressure, Diastolic Blood Pressure, Mean Arterial Pressure, Respiratory Rate, Red Blood Cells, White Blood Cells, Red Blood Cell Distribution Width, Platelets, Hemoglobin, Erythrocyte Specific Volume, Glutathione Transaminase, Blood Urea Nitrogen, Serum Calcium) are replaced with the mean of that variable. Variables with abnormal values are handled by the winsorize method, using the winsor2 command, with 1% and 99% as cut-off points. Missing and abnormal data are processed using the STATA software (Version 17).

### 2.6 Statistical analysis

The normality of continuous variables was assessed by the Kolmogorov-Smirnov test. Continuous variables were expressed as mean ± SD for normally distributed variables, median (IQR) for non-normally distributed continuous variables, and numbers (%) for categorical variables. When analyzing baseline characteristics, continuous variables were compared by T-test or One-Way ANOVA, and categorical variables were compared by Pearson’s χ2 test and Fisher’s test. Potential risk factors were identified by using One-Way Cox Regression analysis, and variables with p-values less than 0.1 were included in Multi-Way Cox Regression analysis to identify independent risk factors for in-hospital mortality. Receiver Operating Characteristic (ROC) analysis was used to assess the predictive ability of Lactate, Albumin, LAR, and SOFA for mortality at 28-d of admission, and the sensitivity and specificity of each index, as well as to calculate the Area Under Curve (AUC). The optimal cut-off value of LAR was determined by the Youden index, and this was used to divide the LAR into high value and low value groups. The unadjusted survival curves were then plotted by the Kaplan-Meier (KM) method, and the two groups of curves were compared using the log Rank test. Finally, we also performed a Subgroup analysis to investigate whether LAR had any effect in different subgroups, including Age, Gender, Race, Acute Kidney Injury, Sepsis, Obesity, Hypertension, Respiratory Failure, Diabetes, Atrial Fibrillation, Heart Failure and Serum Sodium Concentration. All analyses were performed using Free statistics software version 1.6 and the statistical package R 4.1.1. A two-tailed test indicating *P* < 0.05 is considered statistically significant.

## 3 Results

### 3.1 Baseline demographic and clinical characteristics

The baseline characteristics of the 28-d admission survival and non-survival groups are listed in **Table 1**. A total of 539 patients matched the inclusion criteria. That includes 227 (42.1%) females and 312 (57.9%) males. The median age of the patients is 57.0(45.0,71.0) years. There were 329 (61%) whites admitted to the hospital. The 28-d post-admission mortality rate was 9.8%. We noticed that AP non-surviving patients are older (*P* < 0.001) and had lower SBP, DBP, and MBP, while higher RR and SOFA when compared to the 28-d survival group. CRRT and Vasopressin accounted for 50.9% and 58.5% of the treatment management of patients in the non-survival group, and 71.7% had respiratory failure complications, but only 34% of non-surviving patients had combined heart failure. Laboratory indicators showed that LAR at admission was higher in the non-survival group than in the survival group (1.2 [0.6, 1.9] vs 0.6 [0.4, 0.9], *P* < 0.001). And RDW, Serum Creatinine, BUN, PT, INR, Albumin, and Lactate were all remarkably higher (*P* < 0.05) than in the survival group, while the remaining covariates were not significantly different in the two groups (*P* > 0.05).

**Table 1:** Baseline characteristics between survivors and non-survivors.

Variables	Total (N = 539)	28-dsurvivors (N = 486)	28-d non-survivors (N = 53)	*P* - Value
Age (yr)	57.0 (45.0, 71.0)	56.0 (44.0, 69.0)	71.0 (58.0, 81.0)	**< 0.001**
Gender (%)				0.842
Female	227 (42.1)	204 (42)	23 (43.4)	
Male	312 (57.9)	282 (58)	30 (56.6)	
Ethnicity (%)				**0.009**
White	329 (61.0)	304 (62.6)	25 (47.2)	
Black	53 (9.8)	50 (10.3)	3 (5.7)	
Other	157 (29.1)	132 (27.2)	25 (47.2)	
HR (beats/min)	98.7 (85.3, 111.2)	98.6 (85.1, 111.3)	99.4 (86.9, 111.0)	0.947
SBP (mmHg)	119.5 (108.4, 134.2)	120.8 (109.1, 135.2)	110.0 (102.1, 119.6)	**< 0.001**
DBP (mmHg)	66.8 (59.0, 76.3)	67.2 (59.4, 77.6)	64.1 (54.5, 68.5)	**0.002**
MBP (mmHg)	80.3 (72.8, 91.3)	81.3 (73.3, 91.9)	74.6 (70.0, 82.4)	**< 0.001**
RR (beats/min)	21.3 (18.4, 24.6)	21.1 (18.2, 24.5)	23.1 (20.7, 25.7)	**< 0.001**
SOFA	6.0 (3.0, 10.0)	6.0 (3.0, 9.0)	11.0 (8.0, 15.0)	**< 0.001**
Vasopressin, n (%)				**< 0.001**
No	447 (82.9)	425 (87.4)	22 (41.5)	
Yes	92 (17.1)	61 (12.6)	31 (58.5)	
Octreotide, n (%)				0.357
No	506 (93.9)	458 (94.2)	48 (90.6)	
Yes	33 (6.1)	28 (5.8)	5 (9.4)	
Statins, n (%)				0.347
No	409 (75.9)	366 (75.3)	43 (81.1)	
Yes	130 (24.1)	120 (24.7)	10 (18.9)	
Fibrates, n (%)				0.236
No	505 (93.7)	453 (93.2)	52 (98.1)	
Yes	34 (6.3)	33 (6.8)	1 (1.9)	
Metformin, n (%)				1
No	523 (97.0)	471 (96.9)	52 (98.1)	
Yes	16 (3.0)	15 (3.1)	1 (1.9)	
Ventilation, n (%)				0.404
No	59 (10.9)	55 (11.3)	4 (7.5)	
Yes	480 (89.1)	431 (88.7)	49 (92.5)	
CRRT, n (%)				**< 0.001**
No	467 (86.6)	441 (90.7)	26 (49.1)	
Yes	72 (13.4)	45 (9.3)	27 (50.9)	
ERCP, n (%)				0.127
No	526 (97.6)	476 (97.9)	50 (94.3)	
Yes	13 (2.4)	10 (2.1)	3 (5.7)	
AKI (%)				**< 0.001**
No	376 (69.8)	354 (72.8)	22 (41.5)	
Yes	163 (30.2)	132 (27.2)	31 (58.5)	
Sepsis (%)				0.08
No	275 (51.0)	254 (52.3)	21 (39.6)	
Yes	264 (49.0)	232 (47.7)	32 (60.4)	
Obesity (%)				0.853
No	482 (89.4)	435 (89.5)	47 (88.7)	
Yes	57 (10.6)	51 (10.5)	6 (11.3)	
Hypertension (%)				0.87
No	279 (51.8)	251 (51.6)	28 (52.8)	
Yes	260 (48.2)	235 (48.4)	25 (47.2)	
RF (%)				**< 0.001**
No	279 (51.8)	264 (54.3)	15 (28.3)	
Yes	260 (48.2)	222 (45.7)	38 (71.7)	
Diabetes (%)				0.072
No	355 (65.9)	326 (67.1)	29 (54.7)	
Yes	184 (34.1)	160 (32.9)	24 (45.3)	
AF (%)				0.22
No	422 (78.3)	384 (79)	38 (71.7)	
Yes	117 (21.7)	102 (21)	15 (28.3)	
HF (%)				**0.003**
No	437 (81.1)	402 (82.7)	35 (66)	
Yes	102 (18.9)	84 (17.3)	18 (34)	
RBC (m/uL)	3.8 (3.3, 4.4)	3.9 (3.3, 4.5)	3.7 (3.3, 4.3)	0.201
WBC (K/uL)	12.6 (9.0, 18.0)	12.6 (9.1, 18.0)	14.4 (7.6, 17.9)	0.970
RDW (%)	14.5 (13.6, 15.7)	14.4 (13.5, 15.6)	15.2 (14.2, 16.3)	**0.001**
Platelets (K/uL)	195.0 (141.5, 259.0)	196.5 (142.0, 259.5)	192.0 (124.0, 249.0)	0.355
Hemoglobin (g/L)	118.1 ± 24.8	118.7 ± 25.1	112.9 ± 21.6	0.105
Lymphocytes (%)	8.6 (5.0, 14.4)	9.0 (5.0, 14.6)	7.6 (5.0, 11.4)	0.175
Hematocrit (%)	35.8 ± 7.1	35.9 ± 7.2	35.0 ± 6.4	0.378
TBIL (mg/dL)	1.0 (0.6, 2.4)	1.0 (0.6, 2.3)	1.2 (0.6, 2.7)	0.334
ALT (IU/L)	45.0 (23.0, 140.5)	45.5 (23.0, 136.0)	44.0 (23.0, 142.6)	0.766
Glucose (mmol/l)	7.4 (5.8, 10.2)	7.4 (5.8, 10.2)	6.9 (5.6, 9.2)	0.440
Serum creatinine (mg/dL)	1.0 (0.7, 1.8)	1.0 (0.7, 1.6)	1.6 (0.9, 2.7)	**< 0.001**
BUN (mg/dL)	20.0 (12.0, 33.5)	19.0 (12.0, 32.0)	33.0 (17.0, 53.0)	**< 0.001**
AG (mEq/L)	16.0 (13.0, 19.0)	16.0 (13.0, 19.0)	17.0 (13.0, 22.0)	0.176
PT (s)	14.2 (12.7, 16.4)	14.1 (12.6, 16.0)	15.8 (13.5, 19.4)	**0.001**
INR	1.3 (1.1, 1.5)	1.3 (1.1, 1.5)	1.5 (1.2, 1.8)	**0.001**
Potassium (mEq/L)	4.1 (3.6, 4.5)	4.0 (3.6, 4.5)	4.3 (3.8, 5.1)	**0.030**
Sodium (mEq/L)	139.0 (135.0, 142.0)	139.0 (135.0, 141.8)	139.0 (137.0, 143.0)	0.111
Calcium (mg/dL)	8.0 (7.3, 8.6)	8.0 (7.4, 8.6)	7.8 (7.1, 8.5)	0.370
Albumin (g/L)	29.8 ± 6.2	30.1 ± 6.1	26.5 ± 6.5	**< 0.001**
Lactate (mmol/L)	1.8 (1.2, 2.8)	1.7 (1.2, 2.6)	2.8 (1.8, 4.6)	**< 0.001**
LAR (×10^-1^)	0.6 (0.4, 1.0)	0.6 (0.4, 0.9)	1.2 (0.6, 1.9)	**< 0.001**

HR, Heart Rate; SBP, Systolic Blood Pressure; DBP, Diastolic Blood Pressure; MBP, Mean Blood Pressure; RR, Respiratory Rate; SOFA, Sequential Organ Failure Assessment; CRRT, Continuous Renal Replacement Therapy; ERCP, Endoscopic Retrograde Cholangiopancreatography; AKI, Acute Kidney Injury; RF, Respiratory Failure; AF, Atrial Fibrillation; HF, Heart Failure; RBC, Red Blood Cell; WBC; White Blood Cell; RDW, Red Blood Cell Distribution Width; TBIL, Total Bilirubin; ALT, Alanine Aminotransferase; BUN, Blood Urea Nitrogen; AG, Anion Gap; PT, Prothrombin Time; INR, International Normalized Ratio; LAR, Lactate/Albumin Ratio. P - value less than 0.05 is expressed in bold.

### 3.2 The LAR is an independent risk factor for all-cause mortality at 28-d of hospital admission

Covariates with obvious differences in **Table 1** (*P* < 0.05) were included in the univariate COX regression analysis. Results demonstrated that unadjusted LAR was significantly associated with all-cause mortality within 28-d of hospital admission (HR, 1.45; 95% CI, 1.6 - 1.82; *P* < 0.001). Covariates with *P* < 0.1 in **Table 2** and risk factors with potential were then included in multivariate COX regression analyses. **Table 3** presents adjusted analyses of LAR and in-hospital 28-d all-cause mortality in patients with AP using COX proportional risk models. In the model I, LAR was significantly associated with in-hospital 28-d all-cause mortality (HR, 1.46; 95% CI, 1.15 - 1.85; *P* < 0.002) after adjusting for Age, Sex and Race. In a further, after adjusting for Age, Sex, Race, AF, Serum Creatinine, and Serum Sodium in Model II, LAR remained an independent predictor (HR, 1.59; 95% CI, 1.23 - 2.05; *P* < 0.001).

**Table 2:** Univariate COX analysis of risk factors for death within 28-d in patients by logistic regression analysis.

Variables	Univariable COX		
	HR	95% CI	*P* - value Wald’s test
Age (yr)	1.0001	0.9803-1.0204	0.991
Ethnicity (%)			
White	1(REF)		
Black	0.99	0.3,3.27	0.981
Other	1.24	0.71,2.17	0.444
SBP	0.9972	0.9795-1.0152	0.759
DBP	1.0093	0.9828-1.0365	0.497
MBP	1.0039	0.9763-1.0323	0.784
RR	1.03	0.96-1.11	0.446
SOFA	1.04	0.99-1.1	0.141
CRRT	1.45	0.85-2.49	0.176
Vasopressin	1.12	0.65-1.93	0.694
Spesis	0.77	0.44-1.33	0.345
Obesity	1.3	0.55,3.06	0.547
Hypertension	1.5	0.87,2.58	0.142
RF	0.9974	0.547-1.8186	0.993
Diabetes	1.37	0.79-2.36	0.262
AF	0.36	0.2-0.66	**< 0.001**
HF	0.91	0.52-1.61	0.753
RDW	0.98	0.87-1.1	0.738
Serum creatinine	1.14	0.98-1.34	**0.099**
BUN	1.0043	0.994-1.0147	0.415
PT	1.0007	0.9721-1.0302	0.961
INR	1.0078	0.7437-1.3656	0.96
Potassium	1.16	0.92-1.48	0.218
Sodium	1.05	1.01-1.11	**0.029**
Albumin	0.99	0.95-1.03	0.517
Lactate	1.11	1.02-1.21	**0.016**
LAR	1.45	1.16-1.82	**0.001**

SBP, Systolic Blood Pressure; DBP, Diastolic Blood Pressure; MBP, Mean Blood Pressure; RR, Respiratory Rate; SOFA, Sequential Organ Failure Assessment; CRRT, Continuous Renal Replacement Therapy; ERCP, Endoscopic Retrograde Cholangiopancreatography; AKI, Acute Kidney Injury; RF, Respiratory Failure; AF, Atrial Fibrillation; HF, Heart Failure; RDW, Red Blood Cell Distribution Width; BUN, Blood Urea Nitrogen; PT, Prothrombin Time; INR, International Normalized Ratio; LAR, Lactate/Albumin Ratio. P-values less than 0.05 is shown in bold. P < 0.1 is included in the multivariate regression analysis.

**Table 3:** Multivariate COX analysis of risk factors for death in patients within 28-d by logistic regression analysis.

Variables	Multivariable COX			
	HR		95% CI	*P* - value
**Model I**				
LAR		1.46	1.15~1.85	**0.002**
Age		1.01	0.98~1.03	0.563
Gender(male)		1.14	0.65~1.99	0.644
Ethnicity(black)		1.12	0.32~3.91	0.854
Ethnicity(other)		1.10	0.62~1.94	0.746
**Model II**				
LAR		1.59	1.23~2.05	**<0.001**
Age		1.02	0.99~1.04	0.183
Gender(male)		0.94	0.52~1.7	0.845
Ethnicity(black)		0.52	0.13~2.03	0.343
Ethnicity(other)		0.64	0.34~1.2	0.161
AF		0.28	0.14~0.55	**<0.001**
Serum. creatinine		1.12	0.93~1.35	0.217
Sodium		1.09	1.03~1.16	**0.005**

AF, Atrial Fibrillation; LAR, Lactate/Albumin Ratio. P - value less than 0.05 are indicated in bold.

### 3.3 ROC curve analysis and Kaplan–Meier curve

**Figure 2:**
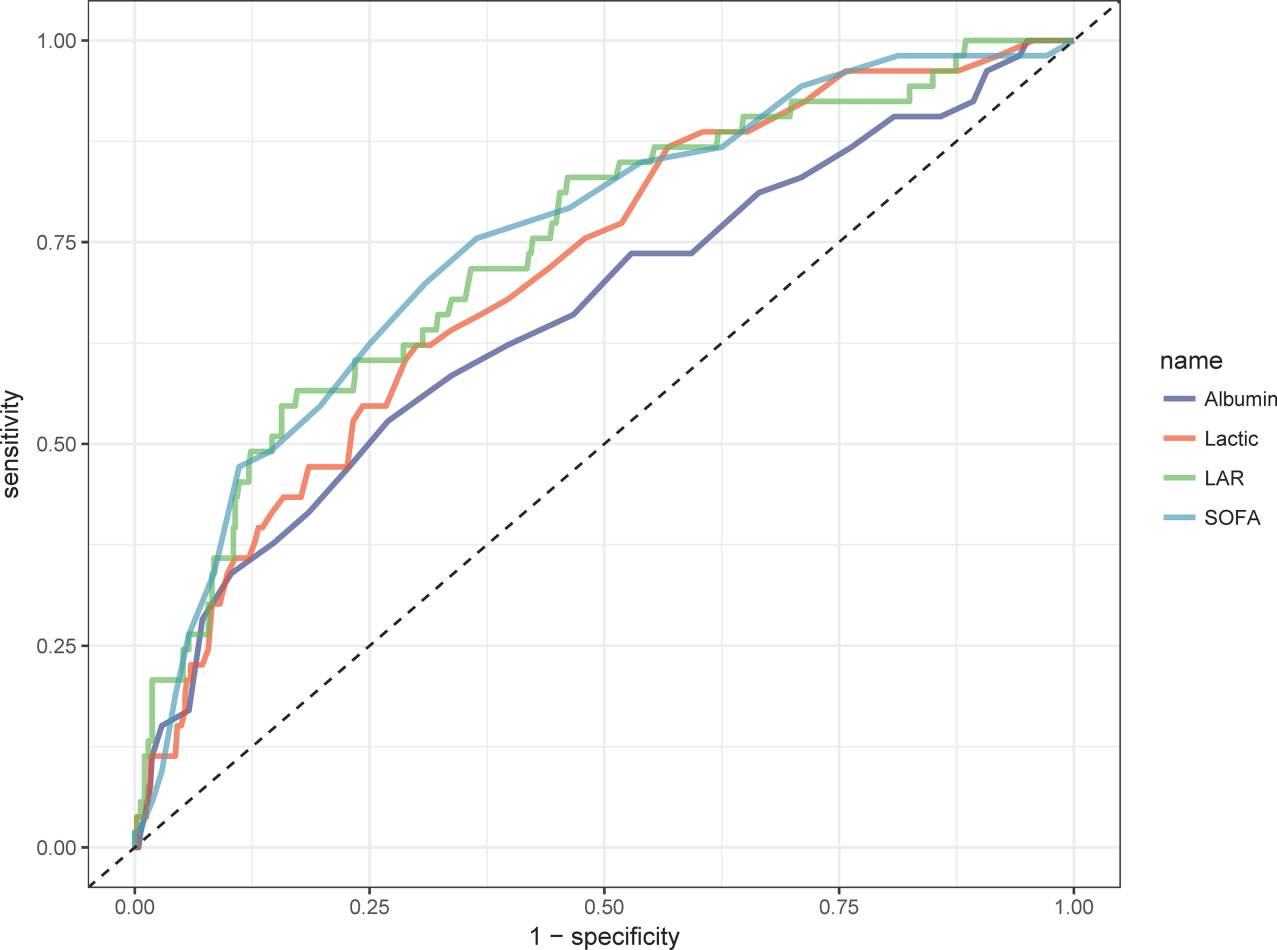
ROC curves of peripheral differential leukocyte count correlate for predicting in-hospital mortality. The green solid line indicates the ROC curve of the LAR. The red solid line indicates the ROC curve for Lactate. Deep blue indicates the ROC curve of Albumin. LAR, lactate/albumin ratio; SOFA, Sequential Organ Failure Assessment.

**Figure 3:**
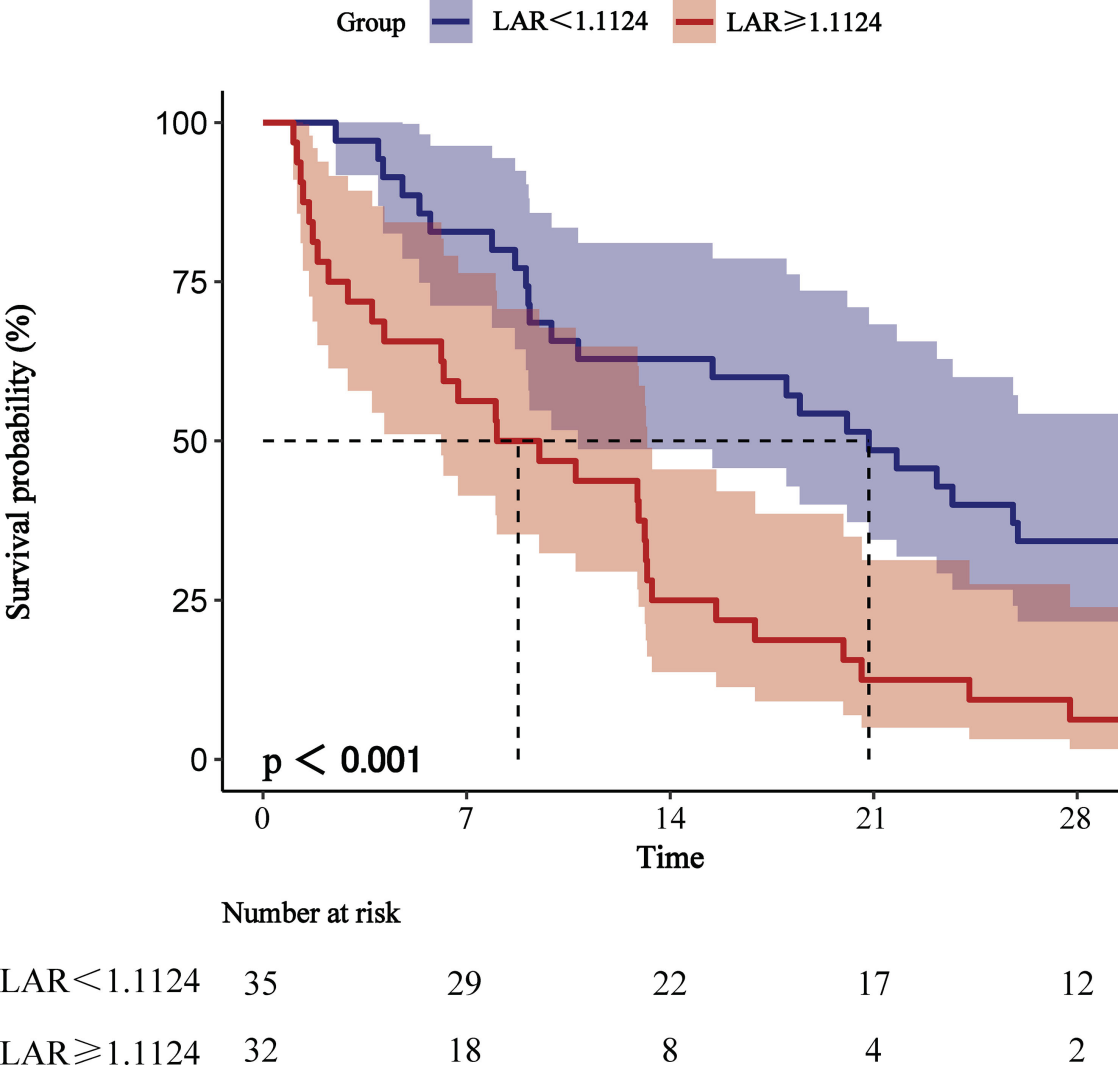
Kaplan-Meier survival analysis curves for all-cause mortality within 28-d of hospital admission.

We plotted ROC curves for the four indicators of LAR, Lactate, Albumin and SOFA for predicting all-cause mortality within 28-d of admission in patients with AP, and the information in **Figure 2** is listed in **Table 4**. The AUC of LAR [74.262% (95% CI: 67.024% - 81.501%)] was obviously superior to Lactate [71.252% (95% CI: 64.102% - 78.401%)] and Albumin [65.917% (95% CI: 57.529% - 74.306%)]. Not even inferior to the SOFA [75.153% (95% CI: 68.208% - 82.099%)]. Consequently, LAR has a significant predictive advantage. We meanwhile obtained the best cut-off value of 1.1124 for LAR, which had a sensitivity of 56.6% and a specificity of 82.7%. According to the optimal cut-off value, AP patients were divided into high admission LAR group (LAR ≥ 1.1124, n = 114) and low admission LAR group (LAR < 1.1124, n = 425). And Kaplan-Meier survival analysis curves were plotted (**Figure 3**), which showed that patients in the high-value group had a significantly higher mortality rate than those in the low-value group (*P* < 0.001).

**Table 4:** Information of ROC curves in **Figure 2**.

Variables	AUC	95%CI	threshold	sensitivity	specificity
LAR	74.262%	67.024% - 81.501%	1.1124	0.566	0.827
Lactate	71.252%	64.102% - 78.401%	2.35	0.6226	0.700
Albumin	65.917%	57.529% - 74.306%	26.5	0.5283	0.731
SOFA	75.153%	68.208% - 82.099%	7.5	0.7547	0.636

AUC, area under the curve; CI, confidence interval; ROC, receiver operating characteristic.

### 3.4 Subgroup analysis

**Figure 4:**
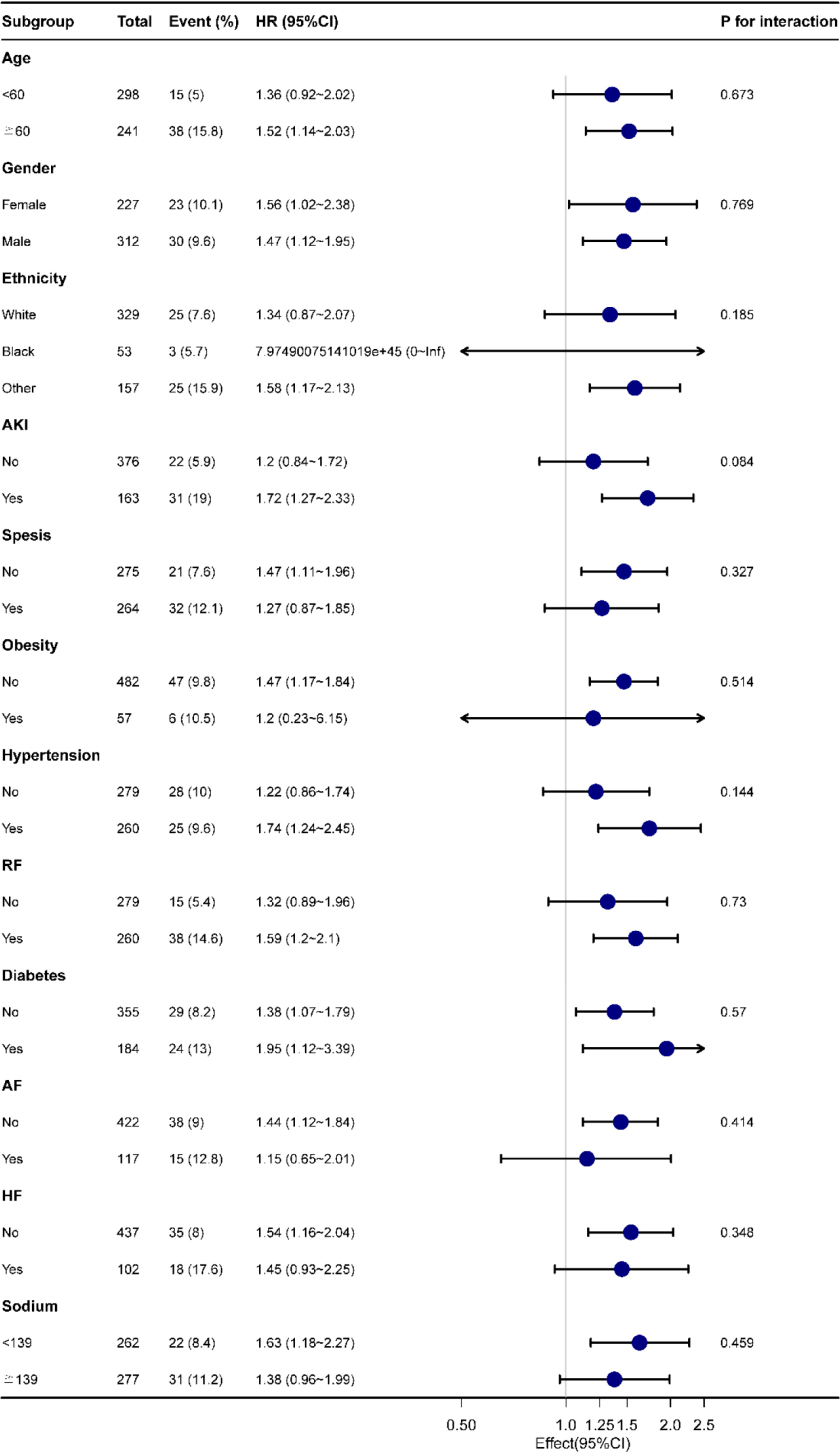
Forest plot for subgroup analysis of the relationship between hospital mortality and LAR. AKI, Acute Kidney Injury; RF, Respiratory Failure; AF, Atrial Fibrillation; HF, Heart Failure.

**Figure 4** indicates as to whether the correlation between LAR and all-cause mortality at 28-d of hospital admission in patients with AP was stable across subgroups. When the stratified analysis was performed for Age, Gender, Race, Race, Acute Kidney Injury, Sepsis, Obesity, Hypertension, Respiratory Failure, Diabetes, Atrial Fibrillation, Heart Failure and Serum Sodium, the forest plot (**Figure 4**) showed no significant interaction of LAR with each subgroup (*P* for interaction: 0.084-0.769). This evidences that LAR is an independent prognostic factor.

## 4 Discussion

The results of this retrospective study demonstrated that the LAR was an independent factor for all-cause mortality in AP patients at 28-d of hospital admission. Comparison by AUC values showed higher accuracy of LAR (74.262%) than lactate (71.252%) or albumin (65.917%) alone. It even compares favourably with SOFA (75.153%). Meanwhile, Kaplan-Meier survival analysis plots indicate that AP patients with LAR ≥1.1124 had significantly higher all-cause mortality within 28-d admissions than those with LAR < 0.001), as well as subgroup analysis further, supports our view.

In recent years, researchers have focused on developing systemic inflammatory biomarkers to predict the prognosis of patients with AP, including RDW (20), Neutrophil/Lymphocyte Ratio (NLR) (21), RDW/Platelet Ratio (RPR) (22), Glycemic/Lymphocyte Ratio (GLR) (23), C-Reactive Protein/Albumin ratio (24), etc. LAR has been applied as a new predictor for Sepsis, Heart Failure and Acute Respiratory Failure (25–27) with a good prediction of mortality. However, studies using LAR to predict the prognosis of AP patients have not been reported.

Lactate being an important indicator of tissue oxygenation, blood perfusion and metabolism *in vivo*, our study illustrated that hyperlactatemia is associated with poor prognosis in critically ill patients, which is consistent with previous research findings (9). Wu et al. similarly identified elevated lactate concentration as an independent risk factor for 28-d mortality in patients with SAP (28). However the interpretation of serum lactate levels is often complex, patients with liver disease can have abnormal lactate metabolism. Furthermore, drugs such as salbutamol and metformin may also cause abnormal elevations of lactate, but this result was not reflected in our study. In addition, some critically ill patients may instead have low lactate levels in venous blood, which reduces the reliability of using lactate alone to predict patient prognosis (29).

The pathogenesis of AP is intimately related to oxidative stress, as activation of the inflammatory response and recruitment of inflammatory cells lead to tissue damage in patients (30). In turn, albumin increases the production of several anti-inflammatory substances such as lipoxins, lysins and protectins to promote wound healing (27), that process consumes large amounts of albumin which could explain the poor prognosis due to reduced albumin levels. It has been reported that serum albumin is a strong predictor of mortality in elderly patients with sepsis (31). However, serum albumin levels are affected by chronic disease, nutritional support and inflammation, which may have limited prognostic value in a single measurement. Now, we make a ratio of blood lactate and serum albumin to predict more accurately the prognosis of AP patients by reducing the effect of a single factor on the regulatory mechanism through the inverse change caused by two different mechanisms (32).

Previously, Cakir et al. (including 1136 patients with an AUC of 0.869 for LAR, 0.816 for lactate, and 0.812 for albumin) results for sepsis studies showed that LAR was a better predictor of in-hospital mortality than the use of lactate or albumin alone (25). Moreover, Shin et al. (including 946 patients) confirmed that LAR was more accurate in predicting mortality in critically ill septic patients at 28-d of admission (15). And these conclusions are in agreement with the outcomes of our study. Thus, monitoring LAR can help to better manage patients with AP in clinical practice.

When patients with AP progress to severe acute pancreatitis (SAP), they will develop hypotension, multi-organ hypoperfusion failure, and adult respiratory distress syndrome due to the activation of the pro-inflammatory cascade response, which is its clinical manifestations similar to those of patients with infectious shock. To some extent, acute pancreatitis can be used as a model for sepsis (33). We included 49% of patients with concurrent sepsis in our study, while the number of patients who died within 28-d of admission was as high as 60.4%. By comparing the optimal cut-off values, our study LAR optimal cut-off value was 1.1124 (because our study albumin units were different), while the Chebl et al. study LAR optimal cut-off value was 0.115, which was nearly the same as our study (29). However, for different etiologies of admission, the optimal cut-off value still needs to be determined by prospective studies.

Of course, there are still some limitations to our study. First, our study is a single-center retrospective cohort study, which cannot elucidate the relationship between LAR and AP as prospective studies do, to the extent that our findings lack some persuasive power. Second, we studied the relationship between LAR and prognosis measured for the first time after hospital admission, which does not allow us to assess the prognostic impact of dynamic LAR. Finally, the population data for our study were from MIMIC-IV (v1.0), which was covering hospitalized patients between 2008-2019. As medical treatment has evolved and therapy regimens have been optimized, such a wide period does not guarantee consistency in treatment regimens for patients admitted to the hospital around that time, which we were unable to determine whether this would have biased the study results.

## 5 Conclusion

In our study, LAR could be used as an independent predictor of all-cause mortality in AP patients within 28-d of admission, which performed better prognostically than blood lactate or serum albumin alone, and was not inferior to SOFA. This will provide healthcare workers with a better tool for timely early intervention and clinic planning for poor patient outcomes. further validation of LAR as a readily available and objective biomarker is still needed in large-scale multicenter prospective studies.

## Data availability statement

The datasets presented in this study can be found in online repositories. The names of the repository/repositories and accession number(s) can be found in the article/supplementary material.

## Ethics statement

The studies involving human participants were reviewed and approved by the Institutional Review Board of the Massachusetts Institute of Technology and Beth Israel Deaconess Medical Center. Written informed consent for participation was not required for this study in accordance with the national legislation and the institutional requirements.

## Author contributions

QL, DL, and HZ were jointly responsible for the conception, design, data collection, data analysis, and editing of the manuscript. MW and JY performed the data processing and data code review. QL and HZ drafted the manuscript. MW and QL reviewed the statistical analysis and literature search. QW and SY revised the manuscript and double-checked the statistical analysis results. All authors made substantial contributions and approved the content of the manuscript.
